# Decision making techniques in mass gathering medicine during the COVID-19 pandemia: a scoping review

**DOI:** 10.3389/fpubh.2024.1493218

**Published:** 2024-10-15

**Authors:** Pedro Llorente-Nieto, José-Manuel Ramos-Rincón, Gregorio González-Alcaide

**Affiliations:** ^1^Conselleria de Sanitat, University of Valencia, Valencia, Spain; ^2^Department of Internal Medicine, Dr. Balmis General University Hospital, Alicante, Spain; ^3^Institute for Health and Biomedical Research (ISABIAL), Alicante, Spain; ^4^Department of Clinical Medicine, Miguel Hernández University of Elche, Alicante, Spain; ^5^History of Science & Documentation Department, University of Valencia, Valencia, Spain

**Keywords:** mass gathering, decision support techniques, COVID-19, disaster medicine, population surveillance

## Abstract

**Background:**

The COVID-19 pandemic has profoundly affected mass gatherings (MGs) worldwide, necessitating the implementation of advanced decision support techniques. These techniques, including mathematical models and risk assessment tools, have played a critical role in ensuring the safe conduct of events by mitigating the spread of SARS-CoV-2.

**Aim:**

This mini-review aims to explore and synthesize the decision support methodologies employed in managing MGs during the COVID-19 pandemic.

**Methods:**

A scoping review was conducted following the PRISMA guidelines covering the period from 2020 to 2024. Studies were categorized by event type (e.g., academic, religious, political, sports) and decision-making tools applied. The review identified a range of decision support techniques, with risk assessment and simulation tools being the most commonly employed across various event types.

**Results:**

A total of 199 studies were initially identified, with 10 selected finally for inclusion based on relevance to decision support techniques. Case studies included the successful risk mitigation strategies during the 2020 Hajj, the 2021 Tokyo Olympics, and the 2022 FIFA World Cup in Qatar. Techniques such as fuzzy logic, Bayesian analysis, and multi-criteria decision-making were also highlighted, particularly in complex scenarios. These tools significantly contributed to reducing COVID-19 transmission risks at large-scale events.

**Conclusion:**

The review underscores the importance of decision support systems in the safe management of MGs during the pandemic. Further research should focus on the integration of emerging technologies and the long-term impacts of decision support tools on public health management.

## 1 Introduction

The COVID-19 pandemic has profoundly affected the organization and management of mass gatherings around the world. Several studies have investigated the implications of SARS-CoV-2 on these events, exploring protective measures, mathematical modeling, and decision support tools to improve safety and a way to improve the level of disaster preparedness at the municipal, provincial, and national levels (1).

Decision support techniques are those mathematical or statistical procedures used as a decision-making aid, which are frequently used in medical decision-making (2). In the specific case of epidemiology and public health surveillance, there are numerous examples of its applications, both in the human and animal kingdoms (3–5).

This mini-review synthesizes the existing literature on decision support techniques in mass gathering medicine during the pandemic, with all the difficulties involved in holding events during this period due to the diversity of opinions and approaches to their safety. Our aim is to provide a comprehensive view of the methodologies adopted to mitigate risks and manage events safely.

## 2 Methods

Our methodology focuses on a short-format scoping review (6) following the PRISMA recommendations (7) for systematic reviews.

### 2.1 Eligibility criteria

A bibliographic search is carried out in Web of Science (WoS) within the Topic field, including the terms “mass gathering” and “COVID” and that are within the period 2020–2024. The search yielded 199 results, with three works discarded due to duplicity. Two reviewers (P.L & J.M.R.R.) independently screened all manuscript titles and abstracts identified in the literature search as potentially relevant. Disagreements were resolved by consulting a third independent reviewer (G.G.A). During the different stages of the selection process, references were managed using Excel. A total of 29 papers are identified that make direct reference in their abstracts to decision support techniques.

### 2.2 Study selection

The three authors of this work independently moved on to the complete reading phase of these 29 works, 19 being discarded in a consensual manner for the following reasons: no reference to MGs or hypothetical MGs (*n* = 7), correspondence, commentary, editorial, opinion or letter (*n* = 6), review (*n* = 3), no article peer-reviewed (*n* = 2); not decision-making technique (*n* = 1) with finally, 10 studies were included in this review.

### 2.3 Data extraction

Data were extracted by one reviewer (P.L.) and verified by the second reviewer (J.M.R.R). Data were extracted using specific marking colors to match the outcomes of interest. MGs can be classified according to the type of event as religious, sports, cultural, political or musical (8). We will develop the selected works more broadly according to the type of event.

### 2.4 Data synthesis and analysis

A narrative synthesis of the selected works is carried out, classifying them according to the decision support tools used. No meta-analysis was performed. We focus our data analysis on identifying, by fully reading the works, the different decision-making techniques, as well as synthesizing the main conclusions of each of the works. We rely on Microsoft^®^ Excel ^®^ to build a table and radial visualization graphs where each of the decision tools are observed being present in the selected works.

## 3 Results

**Figure 1:**
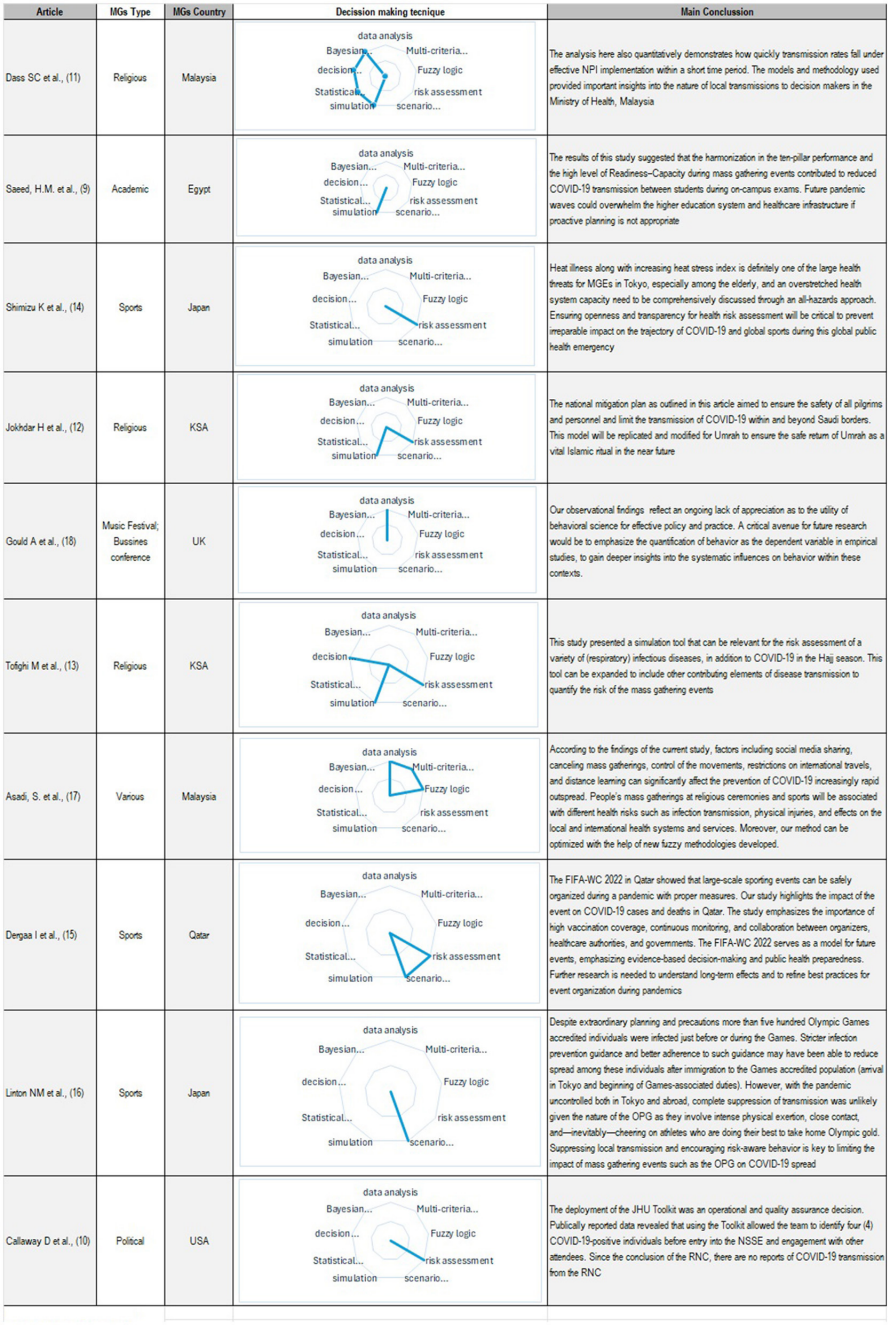
Synthesis of findings.

Ten articles were analyzed, and MGs were categorized according to the type of event studied and the methodologies used in the selected works. Figure 1 provides a summary of 10 documents analyzed. Despite the variety of available techniques, some predominate over others in the selected documents. Notably, some studies stood out for their combination of events and methodologies.

### 3.1 Academic MGs

The study, conducted at the University of October 6 in Egypt (9), assesses the risk of mass gatherings during the COVID-19 pandemic in Egypt, following the WHO Strategic Response Plan. The implementation of intra-action reviews at mass events at the university suggested that the high preparedness reduces the transmission of COVID-19. Coordination among the plan's 10 pillars was crucial to preventing transmission during student meetings.

### 3.2 Political MGs

During the United States Republican Convention (10), the successful use of the Johns Hopkins University risk assessment tool at the Republican National Convention is described, which allowed for the safe holding of the event. These data-driven strategies helped protect communities and the local health system, providing lessons applicable in the reopening of schools and public services.

### 3.3 Religious MGs

In this type of event, we identified three jobs. In the study dedicated to the effect of non-pharmaceutical interventions in Malaysia (11), a heterogeneous SEIR (Susceptible, Exposed, Infected, Removed) model, which is a mathematical model for the study of infectious diseases was used to assess the impact of non-pharmaceutical interventions following the second wave of COVID-19 in Malaysia. The study showed that the motion control command was effective in reducing transmission. Statistical analyses provided relevant information about the local dynamics of the disease and aided in the decision-making of the Malaysian Ministry of Health.

In the Hajj Risk Management work in 2020 (12), Saudi Arabia successfully implemented measures to mitigate COVID-19 risks during the Hajj in 2020, limiting participation to 1,000 pilgrims. No cases of COVID-19 were identified among participants or staff, highlighting the effectiveness of mitigation strategies implemented by the Saudi government to prevent outbreaks at mass events.

Finally, and related to the Hajj, agent-based simulations are used to model risky contacts between pilgrims during the Hajj (13). The results indicated that as the number of pilgrims increased, it was more difficult to maintain physical distancing, suggesting that contact management is key to assessing transmission risks in future events.

### 3.4 Sports MGs

Related to sporting events we find three other works. The first refers to the impact of heat and COVID-19 on the Tokyo 2021 Olympic Games (Japan) (14), where the interaction between the increase in COVID-19 cases and heat illness in Tokyo during the 2021 Olympic Games was analyzed. The authors highlighted that the double burden of COVID-19 and heat-related illnesses could overwhelm health care systems if adequate countermeasures are not put in place.

Secondly, within this type of event we have the assessment of the impact of COVID-19 on the 2022 FIFA World Cup in Qatar (15). This study looked at the impact of COVID-19 during the 2022 FIFA World Cup, revealing a significant increase in cases during the event, but with low mortality rates. The study emphasizes the importance of vaccination and effective collaboration between organizers and health authorities to manage risks at mass events.

Finally, and related to the Tokyo Olympic Games, transmission scenarios are studied through a model of multiple branching processes (16), evaluating the potential transmission of COVID-19 during the Tokyo 2020 Olympic Games. It was estimated that preventive measures could significantly reduce cases, underlining the importance of keeping transmission levels below epidemic levels to avoid contagion between groups.

### 3.5 Other MGs

Our selection of publication highlights two that cannot be classified in the previous points since they address more than one type of MGs.

Malaysia's response to the COVID-19 pandemic (17) used DEMATEL (Decision Making Trial and Evaluation Laboratory) and Fuzzy Rule-Based techniques to assess responses to the pandemic in Malaysia. Movement control orders, international travel restrictions, and the cancellation of mass gatherings were identified as key factors in preventing COVID-19 transmission in the country.

On the other hand, observations of behavior during mass events in Wales (18), an observational study during mass events in Wales showed that personal protective behaviors, such as social distancing and mask wearing, were influenced by the design of the environment and social norms. The results suggest that system-level changes may improve adherence to healthy behaviors in future challenges.

### 3.6 Decision support tools

The most identified decision support tools in our selection of documents have been risk assessment (10, 12–14) which is widely used to assess the potential risks associated with mass gatherings and implement appropriate measures and simulation (9, 11–13) is applied to predict the spread of the virus and assess the impact of different protective measures. To a lesser extent, Bayesian analysis (10), decision support systems (11, 13), statistical modeling (11), data analysis (17, 18), multicriteria decision making (17), fuzzy logic (17) and scenario analysis (16) have been recognized.

## 4 Discussion

The discussion focuses on the current state of decision support techniques and knowledge networks in MGs medicine during the COVID-19 pandemic. We identify research gaps, controversies, and potential future developments in the field.

Regarding research gaps, there are emerging technologies that have been left out of the document selection process, such as blockchain, and that may be interesting to integrate into the management of COVID-19 in mass gatherings (19, 20), in order to track and guarantee compliance with health measures. There is also a need for more exhaustive studies on the long-term impacts of the strategies implemented on public health.

Regarding possible future developments, we recommend: enhanced artificial intelligence powered decision support tools that can provide real-time updates and recommendations and strengthening international collaborations to share data and best practices more effectively.

This work is an initial review of the topic and does not cover many significant works on the topic of interest, e.g. decision making and pandemic management tools developed in Europe. It would be desirable to extend the analysis in the future, to account for more recent and most advanced promising findings.

The study has also a main limitation due to the heterogeneity of the included studies, so interpretation of the results should be taken with caution.

## 5 Conclusion

Our review underscores the critical role of decision support techniques and knowledge networks in managing MGs during the COVID-19 pandemic. By synthesizing existing research and analyzing key collaborations, we provide valuable insights into effective strategies and highlight areas for future research. Continued collaboration and the development of advanced decision support tools are essential to ensure the safety and success of mass gatherings in the post-pandemic era.
